# Normal Incidence Excitation of Out-of-Plane Lattice Resonances in Bipartite Arrays of Metallic Nanostructures

**DOI:** 10.1021/acsphotonics.3c01535

**Published:** 2023-12-18

**Authors:** Juan J. Alvarez-Serrano, Juan R. Deop-Ruano, Vincenzo Aglieri, Andrea Toma, Alejandro Manjavacas

**Affiliations:** † Instituto de Óptica (IO-CSIC), Consejo Superior de Investigaciones Científicas, 28006 Madrid, Spain; ‡ Istituto Italiano di Tecnologia, Via Morego 30, 16163 Genova, Italy

**Keywords:** lattice resonances, periodic arrays, out-of-plane, normal incidence, complex unit cell, non-Bravais lattices

## Abstract

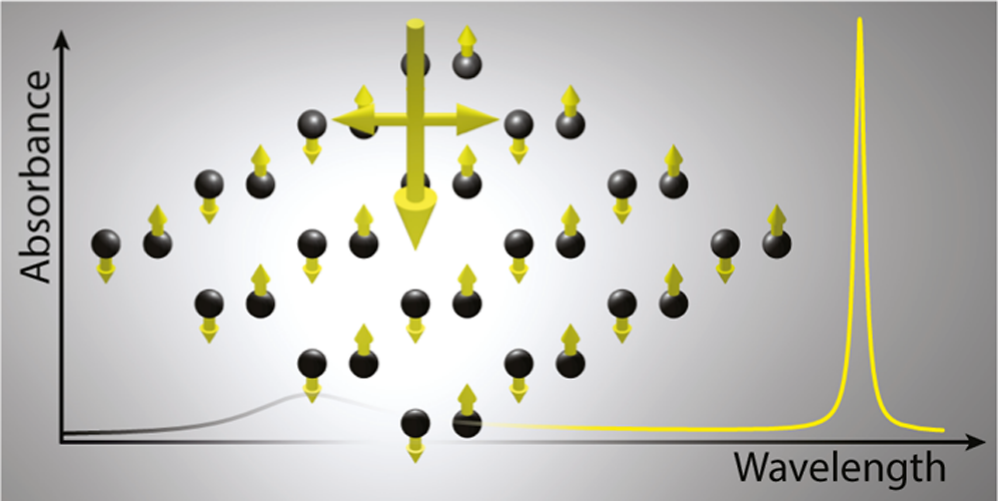

As a result of their coherent interaction, two-dimensional periodic arrays of metallic nanostructures support collective modes commonly known as lattice resonances. Among them, out-of-plane lattice resonances, for which the nanostructures are polarized in the direction perpendicular to the array, are particularly interesting since their unique configuration minimizes radiative losses. Consequently, these modes present extremely high quality factors and field enhancements that make them ideal for a wide range of applications. However, for the same reasons, their excitation is very challenging and has only been achieved at oblique incidence, which adds a layer of complexity to experiments and poses some limitations on their usage. Here, we present an approach to excite out-of-plane lattice resonances in bipartite arrays under normal incidence. Our method is based on exploiting the electric-magnetic coupling between the nanostructures, which has been traditionally neglected in the characterization of arrays made of metallic nanostructures. Using a rigorous coupled dipole model, we demonstrate that this coupling provides a general mechanism to excite out-of-plane lattice resonances under normal incidence conditions. We complete our study with a comprehensive analysis of a potential implementation of our results using an array of nanodisks with the inclusion of a substrate and a coating. This work provides an efficient approach for the excitation of out-of-plane lattice resonances at normal incidence, thus paving the way for the leverage of the extraordinary properties of these optical modes in a wide range of applications.

## Introduction

An ensemble of metallic nanostructures arranged in a periodic pattern is capable of supporting collective modes known as lattice resonances.^1−7^ These modes are the direct consequence of the regular arrangement of the array, which enables the coherent multiple scattering between the optical responses of the individual constituents.^8^ Lattice resonances appear at wavelengths that match the periodicity of the array^9−11^ and, thanks to their collective nature, exhibit strong optical responses with lineshapes much narrower than those associated with the individual nanostructures composing the array.^12−15^ In particular, arrays supporting lattice resonances can reach values of reflectance and absorbance that saturate the theoretical limits^9−11,16^ with quality factors well beyond one thousand.^17−19^ At the same time, they produce very strong near-field enhancements,^20,21^ only limited by the number of elements of the array that are coherently coupled.^22^ As a result of their exceptional properties, lattice resonances are being explored for the development of different optical systems such as color filters,^23,24^ lenses,^25^ light-emitting devices,^26−30^ and chiral elements,^31−34^ as well as ultrasensitive biosensors,^35−38^ light-to-heat transducers,^39,40^ and even platforms to mediate long-range energy transfer,^41−43^ strong coupling,^44,45^ or to achieve Bose-Einstein condensation.^46,47^

Within the field of lattice resonances, those characterized by a dominant polarization oriented perpendicular to the array are particularly interesting.^48−50^ Due to their nature, these modes, which are usually referred to as out-of-plane lattice resonances, cannot efficiently radiate light outside the plane of the array. Furthermore, since the far-field interaction that gives rise to lattice resonances occurs in the directions perpendicular to the polarization,^8^ out-of-plane lattice resonances are inherently more collective than their conventional in-plane counterparts.^43,50^ As a result, these modes display higher quality factors and stronger near-field enhancements.^22^ These properties make out-of-plane lattice resonances ideal for a broad range of applications, such as laser emission^51,52^ and biosensing,^53^ for which they can outperform their conventional in-plane counterparts. Nonetheless, despite their potential, efficient excitation of these modes still remains a challenging task. Due to the transversality condition of electromagnetic radiation, it is typically assumed that out-of-plane lattice resonances in two-dimensional arrays cannot be accessed at normal incidence.^54^ For this reason, previous research efforts have mainly focused on the excitation of these modes at oblique incidence, exploiting the out-of-plane component of the incident electric field.^49,55,56^ However, such a configuration comes with some disadvantages. In particular, it adds a layer of complexity to experiments and limits the use of these modes in integrated devices.

In this work, we propose an approach to excite out-of-plane lattice resonances at normal incidence based on the use of bipartite arrays of metallic nanostructures. In the past, arrays with more than one nanostructure per unit cell have been shown to display interesting optical responses,^15,57−62^ including in-plane lattice resonances with super- and subradiant character.^63,64^ These behaviors were studied and explained by putting the focus on the dominant electric-electric coupling between the different elements of the unit cell, leaving other interaction mechanisms, such as the electric-magnetic one, uninspected. Here, however, we demonstrate how, by exploiting the electric-magnetic coupling between the nanostructures, it is possible to engineer an array capable of sustaining out-of-plane lattice resonances that can be excited under illumination at normal incidence. Using a rigorous coupled dipole model, we show that these modes are associated with strong electric dipoles induced in the two nanostructures of the unit cell, which oscillate perpendicular to the array and in antiphase with one another. Furthermore, we propose and analyze an experimentally feasible implementation of our results using arrays of silver nanodisks placed on a substrate and embedded in a uniform coating. This work provides a general approach to obtaining out-of-plane lattice resonances under normal incidence that can be applied to a wide range of nanostructures and array geometries. Therefore, it constitutes a very promising alternative to more challenging procedures based on the excitation at oblique incidence or the use of nonplanar systems.^65,66^

## Results and Discussion

**Figure 1:**
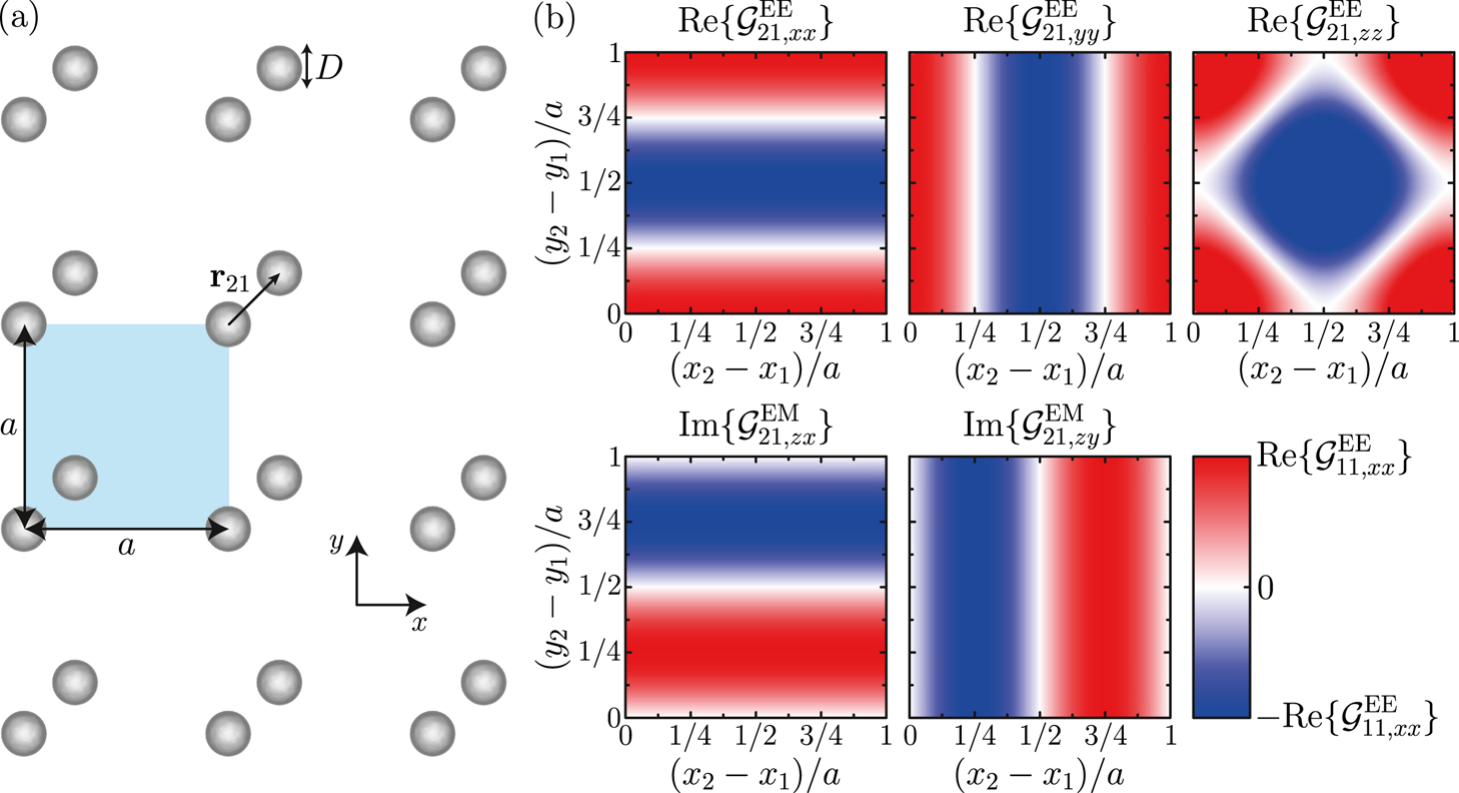
(a) Schematics of the system under consideration, which consists of a square array of period *a* = 800 nm, with two silver nanospheres per unit cell of diameter *D* = 160 nm. (b) Relevant terms of the lattice sum  calculated at the first Rayleigh anomaly (λ = *a*) and normalized to , plotted as a function of the relative position of the nanoparticles in the unit cell.

The system under consideration is depicted in Figure 1a. It is built from the repetition of a unit cell with two silver nanospheres of diameter *D* = 160 nm, over a square lattice with period *a* = 800 nm. We define **T**_μ_ as the vector representing the location of a unit cell μ, and **r**_1_ = (*x*_1_, *y*_1_, 0) and **r**_2_ = (*x*_2_, *y*_2_, 0) as the positions of each particle within the unit cell. Consequently, the absolute location of an arbitrary particle in the array is given by **r**_*i*_ + **T**_μ_, while the relative position between the two constituents of each unit cell is **r**_21_ = **r**_2_ – **r**_1_.

We use a coupled dipole model (CDM)^34,63,67−71^ to study the properties of the periodic array. This method is valid provided that *D* is significantly smaller than both *a* and the wavelength of the light λ. Within this approach, we model each of the nanospheres as a point dipole **d**_*i*_ = (**p**_*i*_, **m**_*i*_) with both electric **p**_*i*_ and magnetic **m**_*i*_ terms. We assume that the array is excited at normal incidence by an electromagnetic plane wave of amplitude **F**_*i*_ = (**E**_*i*_, **H**_*i*_). Although the incident field is the same at the positions of both particles, we retain the subindex for clarity. Then, using Gaussian units, the dipole induced in the *i*-th particle of each unit cell can be expressed as
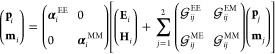
1Here, **α**_*i*_^EE^ and **α**_*i*_^MM^ represent the electric-electric and magnetic-magnetic terms of the polarizability tensor **α**_*i*_ of the *i*-th particle. Notice that due to the symmetry of the nanospheres, the electric-magnetic and magnetic-electric terms satisfy **α**_*i*_^EM^ = **α**_*i*_^ME^ = 0, while **α**_*i*_^EE^ and **α**_*i*_^MM^ are diagonal, i.e.,  and , with  being the 3 × 3 identity matrix. We calculate α_*i*_^EE^ and α_*i*_^MM^ from the dipolar Mie scattering coefficient^72^ using the tabulated dielectric function compiled in ref (73).

The interaction between the particles in the array is described by the lattice sum, which for normal incidence reads , with **G**^ς^(**r**) representing the different terms of the dipole-dipole interaction tensor. The latter are defined as  and **G**^EM^(**r**) = −**G**^ME^(**r**) = *ik*∇ × *e*^*ikr*^/*r*, with *r* = |**r**| and *k* = 2π/λ. The prime symbol in the summation indicates that terms with ν = μ and *i* = *j* are excluded to avoid considering the interaction of a dipole with itself. Notice that the different terms of the lattice sum satisfy  and , as well as  and , a property that will be useful later.

Equation 1 can be solved for the dipole as
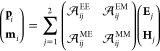
2where  is the ς term of the *i*,*j*-th block of the 12 × 12 tensor , which represents the effective polarizability of the array. This quantity contains all the information on the optical response of the array, arising both from the individual response of the particles, described by **α**, and their interaction, accounted for by the lattice sum .

The objective of this work is to design an array capable of sustaining out-of-plane lattice resonances that can be excited under normal incidence, i.e., using an electromagnetic field that is fully polarized in the *xy*-plane. Consequently, the only possible way to excite out-of-plane modes under such a configuration is by exploiting an interaction capable of mixing the in-plane and out-of-plane components of the response. We know from symmetry arguments that all of the different terms of  (and equivalently ) able to mix the *z*- with the *x*- or *y*-component are zero, which leaves  and  as the only possible candidates to mediate such an interaction.

In Figure 1b, we analyze the relevant terms of  (note that the equivalent terms of  can be readily obtained using the properties listed above). Specifically, each color map shows the strength of the corresponding term as a function of the relative position of the nanoparticles in the unit cell. All of them are calculated at the first Rayleigh anomaly (λ = *a*) and normalized to . Examining these results, we observe that the only terms capable of mixing the in-plane and out-of-plane components are the imaginary parts of . Furthermore, there are four different positions at which these terms reach their extremal values simultaneously. In two of these positions, **r**_21_ = (1, 3, 0)*a*/4 and **r**_21_ = (3, 1, 0)*a*/4, these two terms have the same sign, while in the other two, **r**_21_ = (1, 1, 0)*a*/4 and **r**_21_ = (3, 3, 0)*a*/4, the signs are opposite. Importantly, for any of these positions, all of the EE terms vanish, which allows us to provide a simple explanation for the response of the array. Namely, since only the EM terms survive in such a configuration, the in-plane magnetic dipoles induced by the external magnetic field in the nanoparticles can excite an out-of-plane electric dipole.

**Figure 2:**
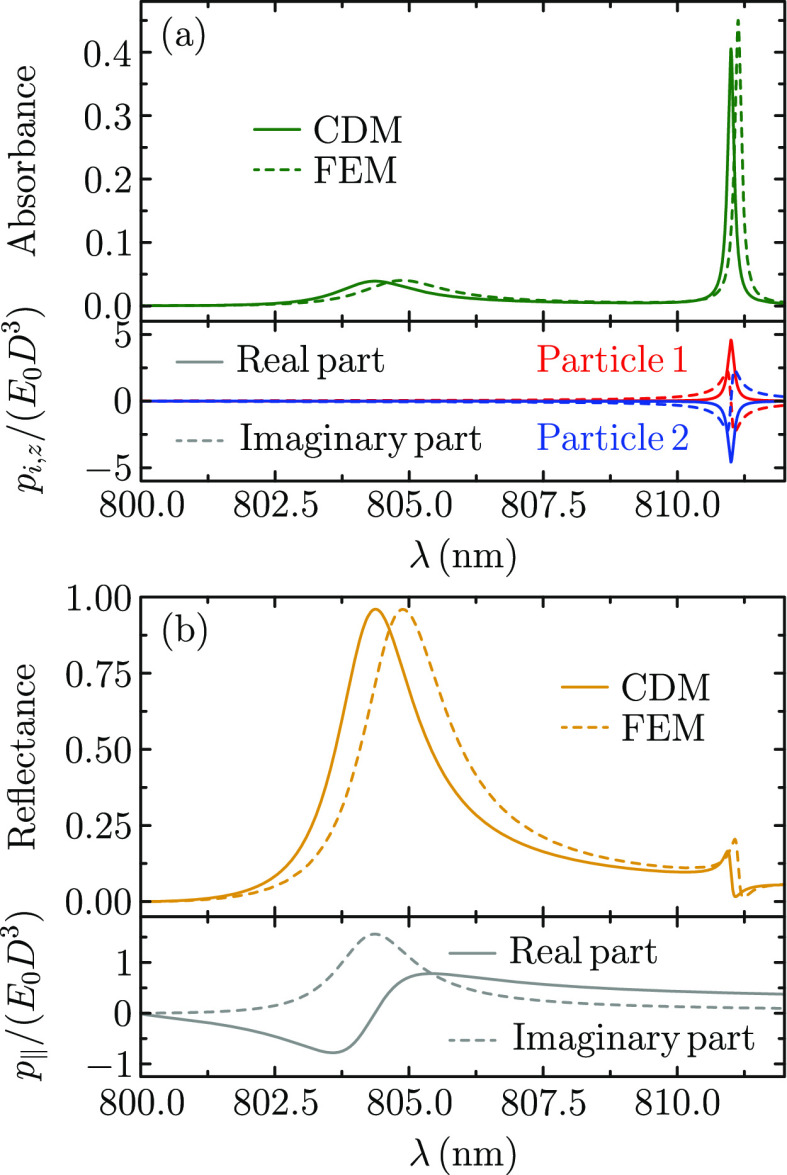
(a) Absorbance spectrum (upper panel) and out-of-plane electric dipole spectra for each of the two particles in the unit cell (lower panel). (b) Reflectance spectrum (upper panel) and in-plane electric dipole spectrum for both particles in the unit cell (lower panel). For comparison, the dashed curves in the absorbance and reflectance spectra show results obtained from FEM simulations.

To maximize the strength of the out-of-plane electric dipole induced in the particles, we place them such that **r**_21_ = (1, 1, 0)*a*/4 and choose an incident field with polarization  and  so that the two EM terms contribute constructively. Notice that we could have equally chosen **r**_21_ = (1, 3, 0)*a*/4 and the orthogonal polarization for the incident field. We analyze the optical response of the resulting array in Figure 2. In particular, the solid curves in the upper panels of Figure 2a,b show, respectively, the absorbance and reflectance spectra of the array. In both cases, we can distinguish two different lattice resonances. The one appearing at lower wavelengths has a relatively broad lineshape with low absorbance and nearly perfect reflectance, while the one located at larger wavelengths displays almost the opposite behavior. In particular, its linewidth is at least 1 order of magnitude smaller and reaches an absorbance over 0.4. These characteristics match the expected behavior of an out-of-plane lattice resonance.

In order to confirm the out-of-plane character of the resonance located at longer wavelengths, we plot, in the lower panel of Figure 2a, the spectrum of the out-of-plane component of the electric dipole for each of the two particles in the unit cell. We normalize their values to *E*_0_*D*^3^ and use red and blue curves for *p*_1,*z*_ and *p*_2,*z*_, respectively, with solid and dashed styles indicating the real and imaginary parts. Examining these results, we observe that, while their values are negligible around the first resonance, the out-of-plane electric dipoles display a sharp feature at the exact spectral position of the second lattice resonance, thus confirming its out-of-plane nature. Indeed, as shown in Figure S1 of the Supporting Information, the spectral position of this mode coincides with the wavelength at which  as expected for the out-of-plane lattice resonance.^43^ Interestingly, both the real and imaginary parts of *p*_1,*z*_ and *p*_2,*z*_ have opposite signs over the entire spectral range under consideration. This behavior, which is ultimately connected to the fact that , provides a subradiant character to the out-of-plane lattice resonance that contributes to further reduce the radiative losses of this mode.

We perform a similar analysis for the in-plane electric dipole in the lower panel of Figure 2b. In this case, due to the symmetry of the array and the polarization of the incident field, both particles display the exact same value of the in-plane electric dipole *p*_∥_ = **p**_*i*_·**E**_*i*_/*E*_0_. This quantity shows a clear peak around λ = 804 nm, matching the position of the first lattice resonance and hence confirming the in-plane character of this mode. Furthermore, since the electric dipoles induced in both particles are exactly equal, this constitutes an example of a superradiant lattice resonance, which explains its large reflectance and small absorbance.^63^ Expectedly, if we excite this system with the orthogonal polarization, the out-of-plane lattice resonance completely disappears from the spectrum. However, in this case, the in-plane lattice resonance displays a small contribution of an out-of-plane magnetic dipole, as analyzed in Figure S2 of the Supporting Information.

To conclude the analysis of the induced dipoles, we compare the roles played by their real and imaginary parts. Usually, the imaginary part of the induced dipole displays a Lorentzian profile,^63^ as in the case of *p*_∥_ plotted in the lower panel of Figure 2b. However, for *p*_1,*z*_ and *p*_2,*z*_, it is the real part that exhibits such a profile, as shown in Figure 2a. This can be explained by recalling that the out-of-plane lattice resonance is excited due to the contributions of the  and  terms of the lattice sum, for which only their imaginary part takes significant values, in contrast with the behavior of all other relevant terms (see colormaps in Figure 1). As a result, an additional imaginary unit is introduced in the response, which ultimately leads to the real part of the dipole exhibiting a Lorentzian profile in the out-of-plane modes.

**Figure 3:**
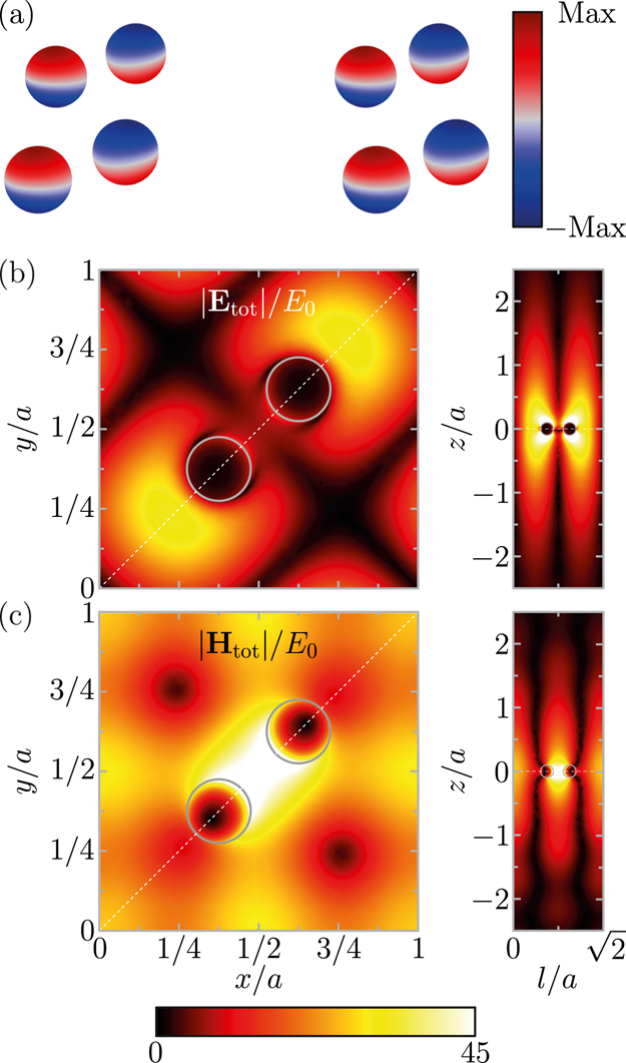
(a) Charge density induced in the particles of four unit cells of the array. (b,c) Total electric (b) and magnetic (c) fields around the array. In both panels, the left column corresponds to the *xy*-plane for a unit cell of the array, while the right column shows a vertical cut along the diagonal of the unit cell signaled by the dashed white line with . The gray contours indicate the positions of the particles. All of the results in this figure are calculated at the wavelength of the out-of-plane lattice resonance from the FEM simulations.

To validate the accuracy of the results shown in Figure 2, we benchmark them against full solutions of Maxwell’s equations obtained by using the finite-element method (FEM). The resulting absorbance and reflectance spectra, which are plotted with dashed curves, are in excellent agreement with the predictions of the CDM, thus confirming the accuracy of our approach. In addition, we can exploit the FEM results to obtain a deeper insight into the properties of the out-of-plane lattice resonance. Specifically, in Figure 3a, we plot the charge induced in the particles at the wavelength of this mode. Exactly as anticipated, the charge distribution corresponds to an out-of-plane dipole with alternating orientations between the two particles in the unit cell.

We also calculate the total electric and magnetic fields around the array, **E**_tot_ and **H**_tot_, which are displayed in Figure 3b,c, respectively. The plots in the left column correspond to one unit cell in the *xy*-plane, while those in the right column show a vertical cut along the diagonal of the unit cell indicated by the white dashed line. The results for the *xy*-plane demonstrate that the out-of-plane lattice resonance produces a large enhancement of the electric field in a considerable region of the unit cell. Notice that because of the opposing orientation of the dipoles induced in the particles, the electric field is almost canceled in the space between them. However, for this same reason, the magnetic field reaches its maximum there with values of enhancement beyond 45. Furthermore, the vertical cuts show that both the electric and magnetic fields of the out-of-plane lattice resonance extend over a distance of almost two periods away from the array. All these results confirm that out-of-plane lattice resonances are advantageous for applications such as lasing^51,52^ and biosensing.^53^ Moreover, the extraordinarily large magnetic fields can be exploited for enhancing processes involving magnetic transitions in molecules.^74−76^

One of the advantages of the CDM, as compared with fully numerical approaches, is that it allows us to dissect the optical response of the array and assess the role played by the different interaction mechanisms. In particular, as stated in eq 2, the dipole induced in the particles of the array is given by , where  is the effective polarizability of the array and **F**_*i*_ = (**E**_*i*_, **H**_*i*_) is the incident electromagnetic field. Therefore, by analyzing the element-wise product , we can identify the dominant contributions to the electric and magnetic dipoles.

**Figure 4:**
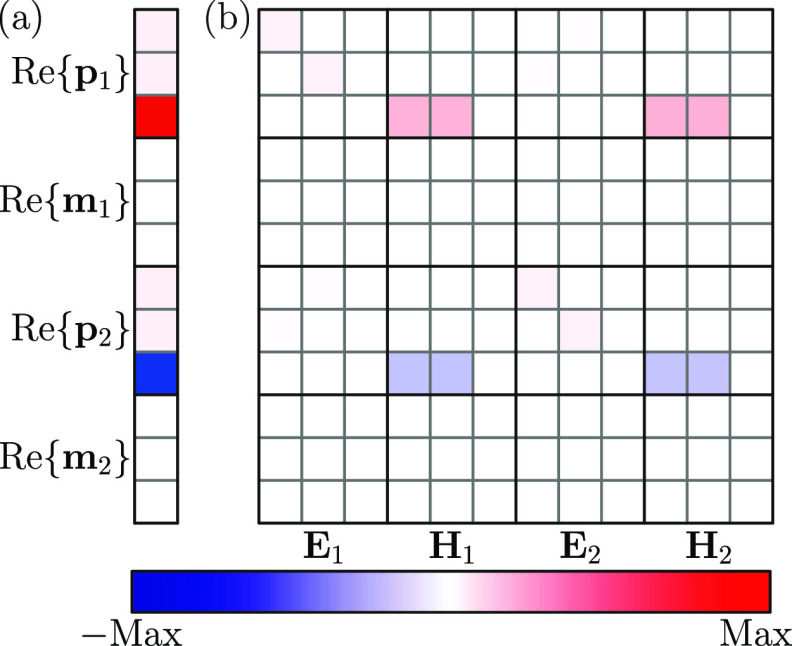
(a) Real part of the electric and magnetic dipoles induced in the two particles of the unit cell. (b) Decomposition of the different contributions to the electric and magnetic dipoles obtained from the element-wise product . All of the results in this figure are calculated at the wavelength of the out-of-plane lattice resonance.

To that end, in Figure 4a, we plot the real part of the electric and magnetic dipoles induced in each of the particles, while in Figure 4b, we show the different terms of the element-wise product . Note that the sum of the elements in each row results in the induced dipole located immediately to their left. We calculate all of these quantities at the wavelength of the out-of-plane lattice resonance and only consider the real parts because, as discussed in Figure 2, the imaginary counterparts are negligible. As expected from our previous characterization, the induced dipoles in both particles are dominated by the out-of-plane electric component and take opposite values in each of them. Analyzing the element-wise decomposition, we conclude that the dominant contribution corresponds to the EM terms. This confirms the central role played by the electric-magnetic coupling in the excitation of the out-of-plane lattice resonance and highlights the bianisotropic nature of the optical response of the array under study. Furthermore, the sign of these terms is consistent with our choice of **r**_21_ and the polarization of the incident field. All of these results are also in accordance with an analysis based on the symmetry of the array,^77,78^ which is detailed in the Supporting Information. Another piece of evidence supporting our interpretation is given in Figure S4 of the Supporting Information, which shows that, if the magnetic response of the particles is neglected, the out-of-plane lattice resonance disappears from the spectrum.

**Figure 5:**
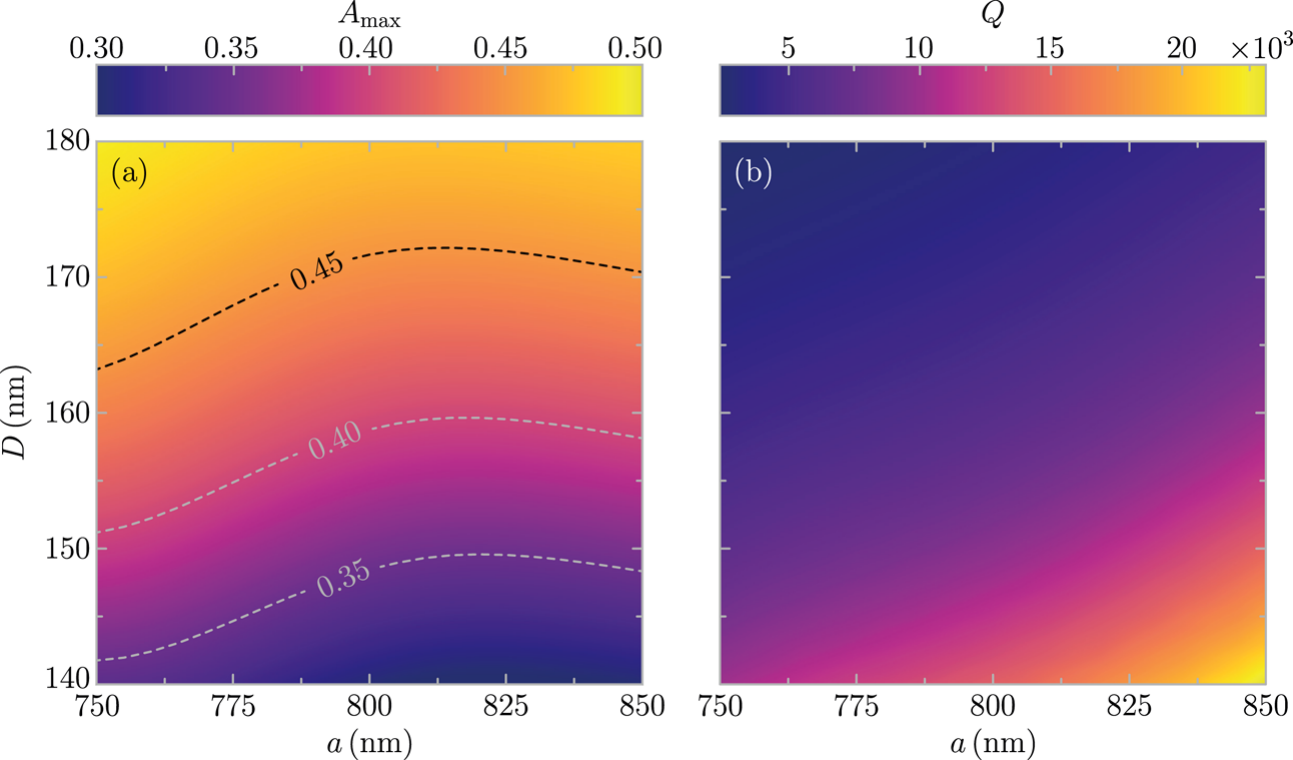
(a) Maximum value of the absorbance *A*_max_ and (b) quality factor *Q* of the out-of-plane lattice resonance as a function of the diameter of the particles *D* and the period of the array *a*.

So far, we have extensively investigated the nature of the out-of-plane lattice resonance. However, to leverage the potential of this mode for applications in nanophotonics, it is very important to fully characterize its optical properties. To that end, in Figure 5a,b, we plot, respectively, the maximum value of the absorbance *A*_max_ and the quality factor *Q* of the out-of-plane lattice resonance as a function of the diameter of the particles *D* and the period of the array *a*. We observe that *A*_max_ monotonically increases with *D*, which is in sharp contrast with the behavior of conventional in-plane lattice resonances.^15,22^ In principle, as the particles become larger, their polarizability and hence their interaction with the incident electromagnetic field increases. However, for in-plane lattice resonances, there is also a decrease of the ratio between the nonradiative and the radiative losses, which ultimately results in a reduction of the absorbance.^22^ On the contrary, since out-of-plane lattice resonances have strongly suppressed radiative losses, the increase in the size of the particles inevitably leads to an enhancement of the absorbance. Importantly, for the larger diameters under consideration, *A*_max_ reaches values close to 0.5, which are highly remarkable for a purely 2D system of metallic structures.

The dependence of *A*_max_ on the period of the array shows a more intricate behavior. For a fixed *D*, *A*_max_ reaches a minimum at a period for which an isocontour, such as those indicated by the dashed curves, becomes horizontal. This nontrivial behavior arises from the interplay between two different mechanisms that produce an increase of the absorbance: smaller periods result in larger filling fractions, while larger periods produce more collective responses.^40^

The quality factor of the out-of-plane lattice resonance, which is analyzed in Figure 5b, has a simpler dependence on *a* and *D*. It monotonically increases with the period and decreases with the size of the particle. This behavior, which is in accordance with previous works on lattice resonances,^34,79^ is associated with the increase of the collective nature of the lattice resonance. It is worth highlighting that the quality factor of the out-of-plane mode takes values in the range of 10^3^ to 10^4^, reaching a value over 2.2 × 10^4^ for the optimum case considered in our calculations.

Throughout this work, we have studied arrays of silver nanospheres surrounded by vacuum since that geometry simplifies the characterization of the optical response of the system. However, an experimental realization of our theoretical predictions requires the presence of a substrate, where the array can be deposited, and a coating that makes the dielectric environment around the array as homogeneous as possible. In addition, particle geometries such as nanodisks are more convenient than nanospheres for common nanofabrication techniques such as electron-beam lithography.

**Figure 6:**
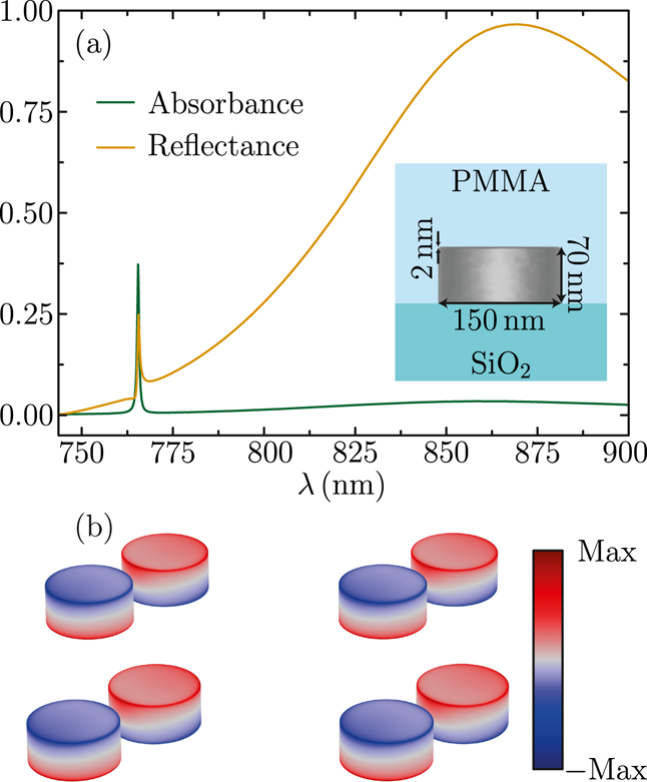
(a) Absorbance and reflectance spectra of an array of silver nanodisks placed on a substrate of fused silica covered with a PMMA coating. The nanodisks have a diameter of 150 nm and a height of 70 nm, and their corners are rounded by a radius of curvature of 2 nm, as shown in the inset. (b) Charge density induced in the nanodisks of four unit cells of the array, calculated at the wavelength of the out-of-plane lattice resonance. All of the results in this figure are obtained from FEM calculations.

Therefore, to facilitate the experimental demonstration of our predictions, in Figure 6, we investigate the optical response of an array of silver nanodisks by using FEM simulations. We consider nanodisks with a diameter of 150 nm and a height of 70 nm, whose corners are rounded by a radius of curvature of 2 nm, as shown in the inset. The nanodisks are placed on top of fused silica and coated with poly(methyl methacrylate) (PMMA), whose dielectric functions we model using the tabulated data of refs (80) and (81), respectively. The relative position between the nanodisks, as well as the polarization of the incident field, is the same as those considered for the array of nanospheres studied before. However, here, the system is excited from the top, i.e., the side of the coating, and we choose a period of *a* = 500 nm to keep the out-of-plane resonance within the visible part of the spectrum.

As shown in Figure 6a, both the absorbance and reflectance spectra display two peaks. Similar to the case of the array of nanospheres, the resonance with a narrow lineshape corresponds to the out-of-plane lattice resonance, while the one with a broad profile is the conventional in-plane mode. However, in this case, the out-of-plane lattice resonance appears at lower wavelengths than the in-plane one. The reason is that nanodisks support in-plane and out-of-plane localized plasmons that appear in the spectrum at different wavelengths depending on their aspect ratio. For our particular choice of parameters, the in-plane localized plasmon of the nanodisks is located at larger wavelengths, very close to the first Rayleigh anomaly. This results in an in-plane lattice resonance that is not very collective and therefore displays a very broad lineshape.^8,79^ The out-of-plane lattice resonance, on the other hand, has a width of the same order as that of the array of nanospheres with a quality factor beyond 10^3^. To complete our characterization of the array of nanodisks, in Figure 6b, we plot the induced charge density at the wavelength of the out-of-plane lattice resonance. As expected, the charge distribution on both nanodisks of the unit cell is indicative of the excitation of out-of-plane dipoles with opposite orientations.

## Conclusions

In summary, we have presented a novel approach to excite out-of-plane lattice resonances at normal incidence in bipartite arrays of metallic nanostructures. Our method exploits the electric-magnetic coupling between the constituents of the array, which has been traditionally neglected in arrays of metallic nanostructures. Using a rigorous coupled dipole model, we have demonstrated that, by choosing the appropriate relative position of the nanostructures in the unit cell, this electric-magnetic coupling provides a mechanism to excite strong out-of-plane electric dipoles from the in-plane magnetic dipoles induced by the external field. This results in the emergence of an out-of-plane lattice resonance, in which the electric dipoles induced in the nanostructures oscillate in antiphase with one another. As a consequence, the out-of-plane lattice resonance displays a large absorbance, with a quality factor beyond 10^4^, and gives rise to exceptionally large enhancements of the electric and magnetic fields in a significant volume around the array. Although we have focused on a square lattice, our approach is general and easily applicable to other geometries such as a triangular lattice, as we demonstrate in Figure S5 of the Supporting Information. Moreover, by using the appropriate polarizability, it can be readily extended to arrays made of other elements such as atoms^82−84^ or dielectric nanostructures.^85^ To complete our study, we have proposed and characterized a potential implementation of our approach that employs an array of nanodisks placed on a substrate and covered by a dielectric coating, thus demonstrating the experimental feasibility of our results. Our work establishes an efficient mechanism to excite out-of-plane lattice resonances at normal incidence in two-dimensional periodic arrays. Therefore, it paves the way to exploit the exceptional quality factors and field enhancements provided by these modes in the development of novel applications in nanophotonics.

## Supporting Information Available

Analysis of the origin of the in-plane and out-of-plane lattice resonances within the coupled dipole model; optical response of the array under consideration for the polarization orthogonal to the one used in Figure 2; analysis of the response of the array under consideration based on its symmetries; analysis of the contribution of the magnetic response to the excitation of the out-of-plane lattice resonance; and excitation of an out-of-plane lattice resonance in a bipartite triangular array under normal incidence (PDF)
