# Supplementary material for: Normal Incidence Excitation of Out-of-Plane Lattice Resonances in Bipartite Arrays of Metallic Nanostructures
Source: ACS Photonics. 2023 Dec 18;11(1):301–9. doi: 10.1021/acsphotonics.3c01535 (PMC10852357; doi:10.1021/acsphotonics.3c01535)
Supplement: Supplementary file 1 — ph3c01535_si_001.pdf [file ph3c01535_si_001.pdf]

Supporting Information: Normal Incidence Excitation of Out-of-Plane Lattice Resonances in Bipartite Arrays of Metallic Nanostructures

Juan J. Alvarez-Serrano,[†,¶] Juan R. Deop-Ruano,[†,¶] Vincenzo Aglieri,[‡] Andrea Toma,[‡] and Alejandro Manjavacas[*,†]

†*Instituto de Óptica (IO-CSIC), Consejo Superior de Investigaciones Científicas, 28006 Madrid, Spain*

‡*Istituto Italiano di Tecnologia, via Morego 30, 16163 Genova, Italy*

¶*Authors contributed equally to this paper*

E-mail: a.manjavacas@csic.es

Table of Contents

- Figure S1 .... Page S-2
- Figure S2 .... Page S-2
- Figure S3 .... Page S-3
- Analysis of the Response of the Bipartite Array Based on its Symmetries .. Page S-3
- Figure S4 .... Page S-5
- Out-of-Plane Lattice Resonance in a Bipartite Triangular Array .... Page S-5
- Figure S5 .... Page S-6

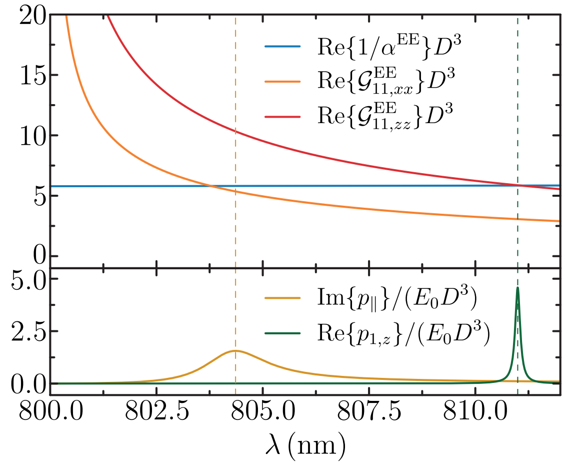

Figure S1: Spectra of the real part of the inverse of α^{EE} and of the in-plane and out-of-plane components of $\mathrm{Re}\{\mathcal{G}_{11}^{\mathrm{EE}}\}$ (upper panel). Spectra of the in-plane and out-of-plane components of the electric dipole for the illumination conditions of Figure 2 of the main paper (lower panel). The vertical dotted lines indicate the maxima of the different electric dipole components.

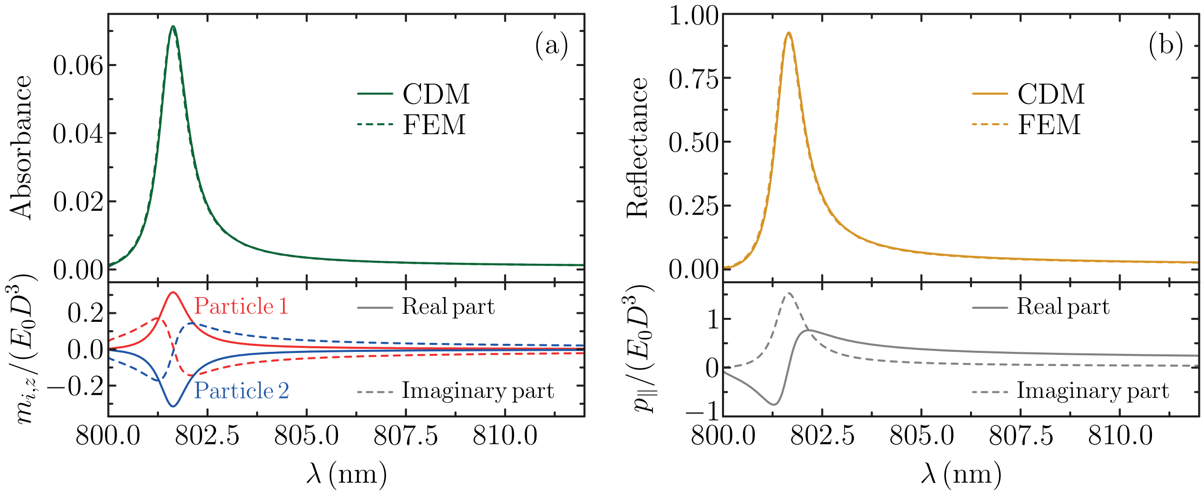

Figure S2: Optical response of the array analyzed in Figure 2 when excited with the orthogonal polarization $\mathbf{E}_i = (1, -1, 0)E_0/\sqrt{2}$ and $\mathbf{H}_i = (1, 1, 0)E_0/\sqrt{2}$. (a) Absorbance spectrum (upper panel) and out-of-plane magnetic dipole spectra for each of the two particles in the unit cell (lower panel). (b) Reflectance spectrum (upper panel) and in-plane electric dipole spectrum for both particles in the unit cell (lower panel). For comparison, the dashed curves in the absorbance and reflectance spectra show results obtained from FEM simulations.

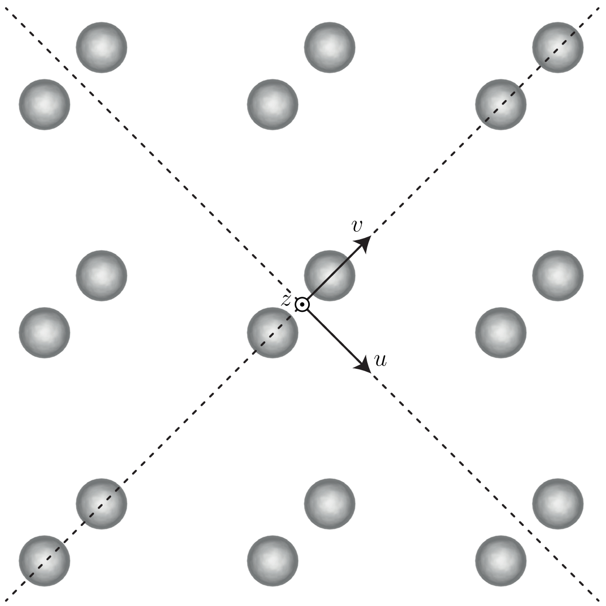

Figure S3: Schematics of the symmetry planes of the bipartite array of Figure 2 of the main paper.

Analysis of the Response of the Bipartite Array Based on its Symmetries.

The bipartite array analyzed in Figure 2 of the main paper is symmetric under reflections with respect to the planes uv, vz, and uz, with $\hat{\mathbf{u}} = (\hat{\mathbf{x}} - \hat{\mathbf{y}})/\sqrt{2}$ and $\hat{\mathbf{v}} = (\hat{\mathbf{x}} + \hat{\mathbf{y}})/\sqrt{2}$, as depicted in Figure S3. Consequently, the effective polarizability of the array $\mathcal{A}$, defined in Equation 2 of the main paper, has to be invariant under such transformations. This is expressed mathematically by

$$\mathcal{A} = \Pi_z \mathcal{A} \Pi_z = \Pi_u \mathcal{A} \Pi_u = \Pi_v \mathcal{A} \Pi_v, \tag{S1}$$

where Π_z, Π_u, and Π_v represent the reflection operators with respect to the plane uv, vz, and uz, respectively. Using the same notation as in the main paper, these matrices are defined

as

$$\Pi_z = \begin{pmatrix} +\sigma_z & 0 & 0 & 0 \\ 0 & -\sigma_z & 0 & 0 \\ 0 & 0 & +\sigma_z & 0 \\ 0 & 0 & 0 & -\sigma_z \end{pmatrix}, \quad \Pi_u = \begin{pmatrix} +\sigma_u & 0 & 0 & 0 \\ 0 & -\sigma_u & 0 & 0 \\ 0 & 0 & +\sigma_u & 0 \\ 0 & 0 & 0 & -\sigma_u \end{pmatrix}$$

and

$$\Pi_v = \begin{pmatrix} 0 & 0 & +\sigma_v & 0 \\ 0 & 0 & 0 & -\sigma_v \\ +\sigma_v & 0 & 0 & 0 \\ 0 & -\sigma_v & 0 & 0 \end{pmatrix}.$$

Notice that the negative signs in these matrices are a consequence of the pseudovector nature of the magnetic dipole and field. Furthermore, σ_z, σ_u, and σ_v are 3×3 matrices, which in the basis formed by the vectors $\hat{\mathbf{u}}$, $\hat{\mathbf{v}}$, and $\hat{\mathbf{z}}$, read

$$\sigma_z = \begin{pmatrix} +1 & 0 & 0 \\ 0 & +1 & 0 \\ 0 & 0 & -1 \end{pmatrix}, \quad \sigma_u = \begin{pmatrix} -1 & 0 & 0 \\ 0 & +1 & 0 \\ 0 & 0 & +1 \end{pmatrix}, \quad \sigma_v = \begin{pmatrix} +1 & 0 & 0 \\ 0 & -1 & 0 \\ 0 & 0 & +1 \end{pmatrix}.$$

Equation S1 imposes that the EE and MM terms of the effective polarizability of the array are diagonal, while for the EM and ME terms, only the components uz and zu take finite values. This is in perfect accordance with the results of Figure 4(b) of the main paper. Moreover, Equation S1 also implies that $\mathcal{A}_{11}^{\varsigma} = -\mathcal{A}_{22}^{\varsigma}$ and $\mathcal{A}_{12}^{\varsigma} = -\mathcal{A}_{21}^{\varsigma}$ for $\varsigma =$ EM and $\varsigma =$ ME, which is consistent with $p_{1,z}$ and $p_{2,z}$ having opposite signs.

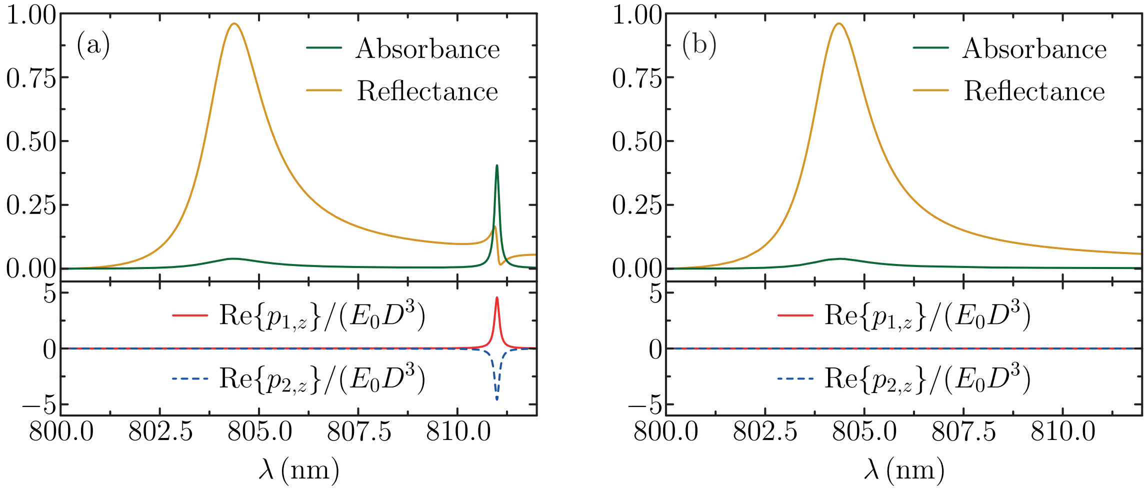

Figure S4: Analysis of the contribution of the magnetic response to the excitation of the out-of-plane lattice resonance. (a) Absorbance and reflectance spectra of the array of Figure 2 of the main paper (upper panel) and the corresponding out-of-plane electric dipole for each of the two particles in the unit cell (lower panel). (b) Same as (a) but neglecting the magnetic response of the particles. Clearly, the out-of-plane lattice resonance only appears in the spectrum when the magnetic response is considered.

Out-of-Plane Lattice Resonance in a Bipartite Triangular Array.

To demonstrate that our approach to obtain out-of-plane lattice resonances under normal incidence excitation is general, we apply it to a bipartite triangular array. We consider an array defined by the lattice vectors $\mathbf{a}_1 = (1,0,0)a$ and $\mathbf{a}_2 = (1,\sqrt{3},0)a/2$, with $a = 850$ nm, which contains two silver nanospheres of $D = 140$ nm per unit cell separated by $\mathbf{r}_{21}$, as shown in Figure S5(a). Following the same steps as in main paper, in Figure S5(b), we plot the relevant terms of $\mathcal{G}^{\varsigma}_{21}$ calculated at the Rayleigh anomaly ($\lambda = \sqrt{3}a/2$), as a function of $\mathbf{r}_{21}$. We choose to excite the system at normal incidence with an electromagnetic plane wave of amplitude $\mathbf{E}_i = (0,1,0)E_0$ and $\mathbf{H}_i = (-1,0,0)E_0$. For such polarization, the optimum position of the particles is $\mathbf{r}_{21} = (1, 0.258, 0)a$, since at that position $\mathrm{Im}\{\mathcal{G}^{\mathrm{EM}}_{21,zx}\}$ is maximized. This term provides the necessary interaction to give rise to the out-of-plane lattice resonance.

The optical response of the array is analyzed in Figures S5(c) and S5(d), demonstrating that this system can support an out-of-plane lattice resonance for normal incidence excitation.

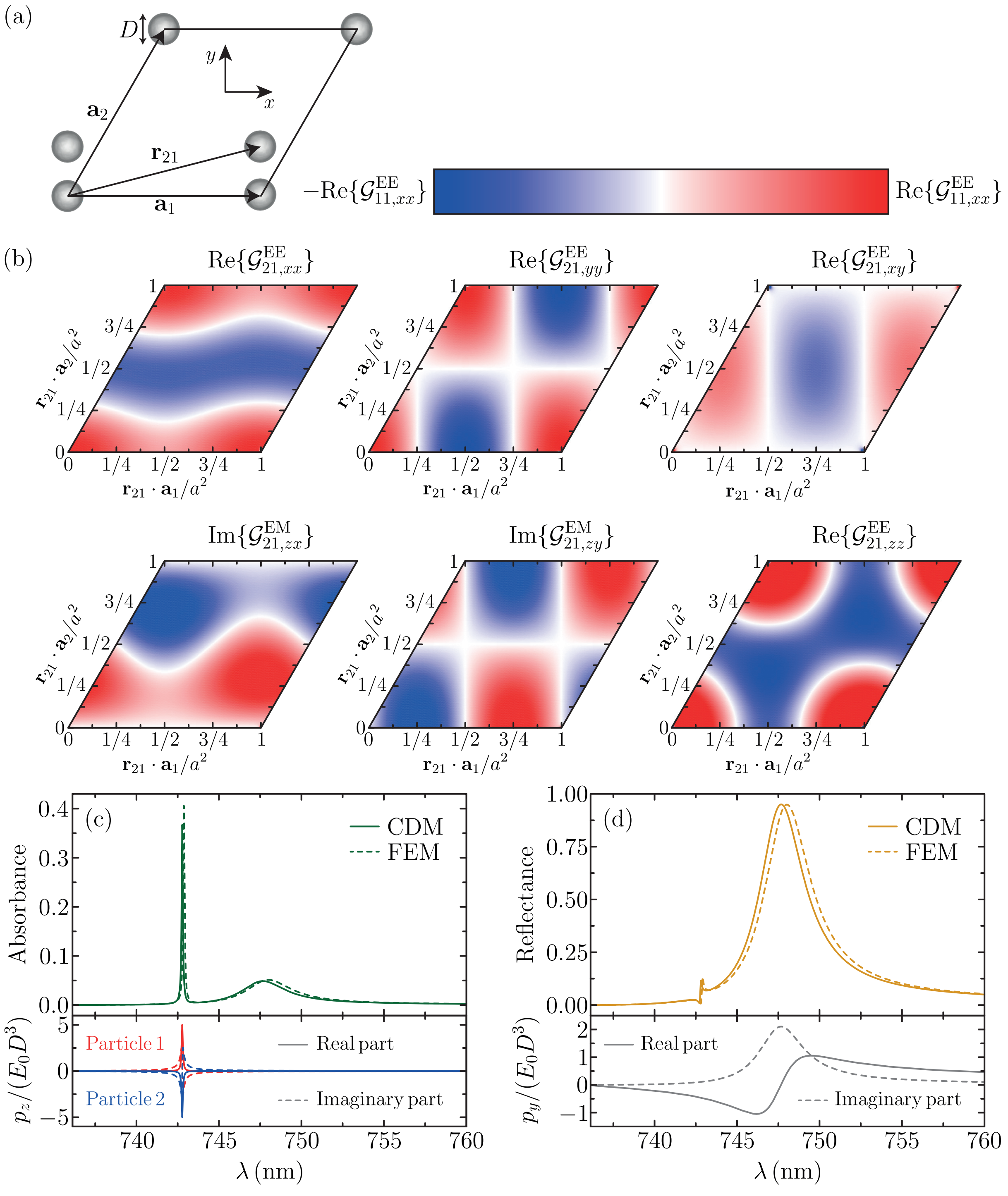

Figure S5: (a) Scheme of the unit cell of the triangular array. (b) Relevant terms of the lattice sum $\mathcal{G}_{21}$ calculated at the first Rayleigh anomaly ($\lambda = \sqrt{3}a/2$) and normalized to $\mathrm{Re}\{\mathcal{G}^{\mathrm{EE}}_{11,xx}\}$, as a function of $\mathbf{r}_{21}$. (c) Absorbance spectrum (upper panel) and out-of-plane electric dipole spectra for each of the two particles in the unit cell (lower panel). (d) Reflectance spectrum (upper panel) and in-plane electric dipole spectrum for both particles in the unit cell (lower panel). For comparison, the dashed curves in the absorbance and reflectance spectra show results obtained from FEM simulations.
